# Photodynamic therapy for caries management: mechanisms, applications, and perspectives

**DOI:** 10.3389/fcimb.2026.1913153

**Published:** 2026-09-14

**Authors:** Qi Li, Peijia Zheng, Hongyan Zhang, Huimin Tao, Xueying Huang, Haiyun Luo, Wenjing Liu

**Affiliations:** Stomatological Hospital, School of Stomatology, Southern Medical University, Guangzhou, China

**Keywords:** antimicrobial, caries, photodynamic therapy, pulp, remineralization

## Abstract

Dental caries poses a severe threat to oral health. However, conventional mechanical and chemical therapies have notable limitations, including incomplete eradication of dental plaque biofilms, difficulty in eliminating deep-seated bacteria, and the emergence of antimicrobial resistance. Photodynamic therapy (PDT), a therapeutic strategy with highly efficient broad-spectrum antimicrobial activity, is widely used to treat oral infections. Notably, PDT has tremendous potential for the prevention and treatment of dental caries, particularly by disrupting cariogenic biofilms. Furthermore, with continued advancement, PDT shows strong potential to promote remineralization and repair of dental hard tissues. However, current reviews on PDT for dental caries fail to provide an in-depth analysis of the biological mechanisms or comprehensively map the mechanistic chain of PDT in caries prevention and management. This review focuses on elucidating how PDT precisely disrupts the progression of cariogenic biofilms across various pathological stages of caries development while also exploring its therapeutic potential to induce dental tissue remineralization and repair. It aims to provide insights into further research and clinical use of PDT against dental caries and to present prospects for developing new targeted photosensitizers and adjusting light parameters to improve caries prevention and management.

## Graphical Abstract

**Figure:**
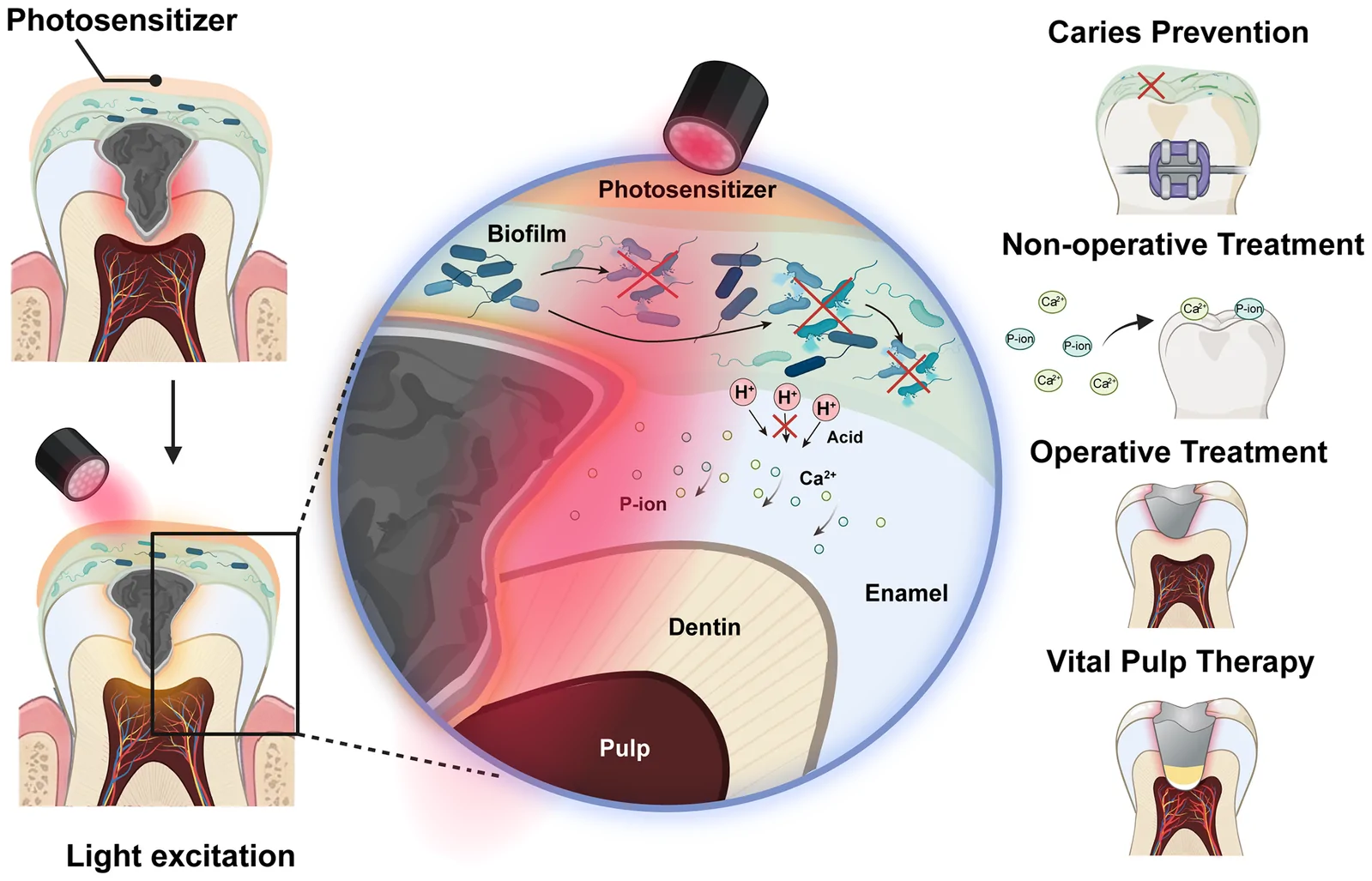

## 1 Introduction

Dental caries is a chronic infectious disease caused by cariogenic biofilm formation. The pathological basis of dental caries is the formation of dense biofilms on tooth surfaces by cariogenic bacteria, primarily *Streptococcus mutans*, *Lactobacillus* spp., and *Actinomyces* spp., within a matrix of extracellular polysaccharides (EPS), food debris, and salivary proteins. These biofilms continuously damage hard dental tissues by metabolizing dietary sugars to produce acids (Pitts et al., 2017). The EPS barrier, strong acidogenicity, acid tolerance, and complex microenvironmental gradients make it difficult to eradicate cariogenic biofilms, which constitute a key pathological basis for the persistently high prevalence of dental caries (Sbordone and Bortolaia, 2003; Koo et al., 2017).

Traditional chemical antimicrobials used in clinical caries management, such as chlorhexidine and cetylpyridinium chloride, can suppress the growth of planktonic cariogenic bacteria but show limited biofilm penetration and are ineffective at fully eradicating mature cariogenic biofilms (Zaura-Arite et al., 2001). Long-term use may cause oral dysbiosis, tooth staining, oral mucosal irritation, and taste disturbances (James et al., 2017). Moreover, increased antimicrobial exposure can promote resistance in cariogenic bacteria, further compromising clinical efficacy and disrupting oral microbial homeostasis (Bescos et al., 2020; Brookes et al., 2021). These limitations prevent traditional chemical antimicrobials from meeting the core clinical needs of caries treatment, highlighting the need for alternative therapeutic strategies.

Photodynamic therapy (PDT) is a noninvasive antimicrobial approach based on the synergistic action of a photosensitizer (PS), a specific light source, and oxygen (Gholami et al., 2022; Li et al., 2023). Its minimally invasive, efficient, and safe nature has supported its broad application and systematic evaluation in oral and maxillofacial infections, periodontitis, peri-implantitis, and endodontic biofilm disinfection (Bumb et al., 2014; Moreira et al., 2015; Plotino et al., 2019; Stájer et al., 2020; Gholami et al., 2022; Wang et al., 2024; Yan et al., 2025). Recent experimental and clinical evidence also indicates substantial potential for PDT in caries prevention and management, particularly through the disruption of cariogenic biofilms (Garcia et al., 2021; Silva Teófilo et al., 2021; Aghili et al., 2023). However, existing reviews have rarely provided an in-depth analysis of the underlying biological mechanisms or a complete mechanistic account of PDT across the entire continuum of caries prevention and treatment. As PDT continues to evolve, it may promote dental hard tissue remineralization and repair. Therefore, a review bridging fundamental mechanisms with clinical applications is warranted to comprehensively outline the current landscape and future perspectives of PDT in the holistic management of dental caries.

This review focuses on the efficacy of PDT in blocking cariogenic biofilm evolution throughout caries progression and highlights its emerging role in fostering tissue repair. We identified two core mechanisms: (1) inhibition of cariogenic biofilm formation and (2) promotion of remineralization and tissue repair. Building on these mechanistic insights, we summarize the current clinical applications of PDT in comprehensive caries management, including caries prevention, non-operative treatment, operative treatment, and vital pulp therapy. Finally, we provide a prospective outlook on the development of novel targeted PSs and the optimization of irradiation parameters to enhance the efficacy of caries management. Our objective is to establish a theoretical foundation that deepens the clinical understanding and refines strategies for therapeutic applications.

## 2 Components of photodynamic therapy

PDT relies on three key components: a PS, a light source, and molecular oxygen, and exerts antimicrobial effects through synergistic Type I and Type II photochemical reactions. Upon excitation, the PS transitions from the ground state (S_0_) to the excited triplet state (T_1_), initiating two pathways. Type I reactions generate free radicals, including hydroxyl radicals (^·^OH), through electron or hydrogen transfer (Lima and Reis, 2023; Yan et al., 2023; Zhu et al., 2026). Type II reaction produces highly reactive singlet oxygen (^1^O_2_), a key mediator of biofilm disruption that oxidatively damages cariogenic bacteria and biofilm components, thereby compromising biofilm structural integrity (Lee et al., 2012; Misba et al., 2018). Together, these reactions provide comprehensive antimicrobial activity against cariogenic biofilms.

### 2.1 Light

In PDT for the treatment of caries, an excitation light source is the core component. The PS can effectively disrupt cariogenic biofilms only after absorbing photons that match its absorption spectrum (Nagata et al., 2012; Paschoal et al., 2024). Achieving antimicrobial activity while ensuring pulpal safety and optimal therapeutic efficacy depends not only on the appropriate choice of light-source hardware but also on the control of physical parameters, including wavelength, fluence, and irradiation mode.

Optimizing light sources is essential for the safe and effective use of PDT in caries treatment. Early PDT systems relied on broad-spectrum, noncoherent sources, such as halogen and xenon lamps, which have largely been abandoned because of their low energy-conversion efficiency and the risk of irreversible thermal pulpal injury (Lee et al., 2012; Nagata et al., 2012). Advances in optical technology have introduced lasers, particularly diode lasers, which enable the precise matching and efficient excitation of specific PSs through their monochromaticity, high coherence, and favorable targeting properties (Konopka and Goslinski, 2007; Cieplik et al., 2017; Moradi et al., 2024). To address the high cost, limited portability, and potential local thermal effects of high-energy lasers, low-power diode lasers can be used to optimize these parameters (Nagata et al., 2012). LED-based PDT has also been widely adopted because of its precise wavelength, minimal heat accumulation, affordability, and portability, which align with the safety requirements of modern minimally invasive dentistry (Mirzaie et al., 2016; Takeuchi et al., 2023). However, its limited tissue penetration makes LED-mediated PDT less effective than laser sources in completely disrupting deep, dense cariogenic biofilms.

The clinical efficacy of PDT depends on the selection of an appropriate light source and precise control of the irradiation parameters. The excitation wavelength must closely match the absorption peak of the PS while balancing tissue penetration and thermal safety, and the wavelength for caries treatment is generally maintained below 810 nm (Nagata et al., 2012; Asnaashari et al., 2016; Shiotsu-Ogura et al., 2019). The power density and total fluence should be adjusted downward according to the thickness of the residual dentin between the carious lesion and pulp to avoid irreversible thermal pulpal injuries (Melo et al., 2015; Mirzaie et al., 2016). An ideal PDT light source should match the absorption spectrum of the PS, provide sufficient intensity for activation without thermal damage, and deliver appropriate fluence to the target tissue.

### 2.2 Photosensitizers

PSs are another key component of PDT (Zhao et al., 2024). A PS is only effective when it absorbs light from a suitable source. As the wavelengths used for PDT in caries treatment generally do not exceed 810 nm, suitable PSs should have absorption maxima within this range (Nagata et al., 2012; Asnaashari et al., 2016). The PSs currently used for caries prevention and treatment can be classified into the following categories.

Phenothiazinium PSs are the most established group of antimicrobial PSs used in caries-related PDT. The main representatives, methylene blue (MB) and toluidine blue (TBO), are activated at 630–660 nm (Cieplik et al., 2017; Saffarpour et al., 2022; AlSunbul and Murriky, 2023). Both are water-soluble cationic PSs with strong activity against gram-negative (G^−^) bacteria (Usacheva et al., 2003).

The second class, xanthene PSs, includes Rose Bengal and erythrosine. They are primarily activated by green or blue light at 500–550 nm and are well suited for LED excitation (Ishiyama et al., 2012; Ahrari et al., 2023). Clinically, they may also serve as plaque-disclosing agents, combining plaque visualization and antimicrobial activity.

The third class, cyanine dyes, is primarily represented by indocyanine green (ICG). Its absorption maximum lies in the near-infrared region, typically at 800–808 nm, making it the PS with the longest absorption wavelength currently used in PDT for caries treatment. Therefore, it is compatible with laser light sources (Aytac Bal et al., 2019; Alkhudhairy, 2025).

The fourth class, chlorins and their emerging derivatives, comprises primarily chlorin e6 (Ce6) and Fotoenticine (FTC). They are typically activated near 660 nm using laser light sources (Garcia et al., 2021; Wang et al., 2023).

The fifth class, naturally derived PSs, comprises curcumin, riboflavin, and phycocyanin. Curcumin and riboflavin are activated by blue light at 400–500 nm and are well suited for LED excitation (Ahrari et al., 2023, 2024). Phycocyanins are activated at approximately 635 nm (Moradi et al., 2024). All these PSs possess excellent biocompatibility and leave no chemical residues, making them well suited for caries treatment (Kamran et al., 2022; Kashi et al., 2025).

Although conventional PSs can produce effective photodynamic effects, high concentrations may promote dimer formation and reduce photodynamic efficiency, and many PSs have limited water solubility. Nanotechnology has helped to overcome these limitations. Encapsulation in nanostructures, such as liposomes and polymer-based nanoparticles, improves water solubility and suppresses molecular dimerization, thereby preserving photodynamic activity. Alternatively, nanomaterials, such as graphene and fullerenes, can act directly as PSs. Both approaches may further enhance PDT performance (Qi et al., 2019; Pham et al., 2021; Yan et al., 2023). The emergence of PSs that promote mineralization and repair may also enable PDT to combine antibacterial activity with the induction of dental hard tissue mineralization, offering additional opportunities to improve clinical outcomes in caries treatment (Lu et al., 2023; Erckmann et al., 2025).

## 3 Mechanisms of PDT in inhibiting caries progression

### 3.1 PDT inhibition of cariogenic biofilm formation

The formation of cariogenic biofilms is a complex and orderly process that begins with bacterial attachment to tooth surfaces. This is followed by bacterial aggregation and proliferation, along with the production of EPS and other components, ultimately leading to the development of a mature biofilm structure (Kolenbrander et al., 2010; Marsh, 2010; Huang et al., 2011). PDT disrupts this process through Type I and Type II photochemical reactions (Misba and Khan, 2018; Li et al., 2023). PDT prevents dental caries by limiting bacterial colonization, biofilm formation, and acid production (**Figure 1**).

**Figure 1:**
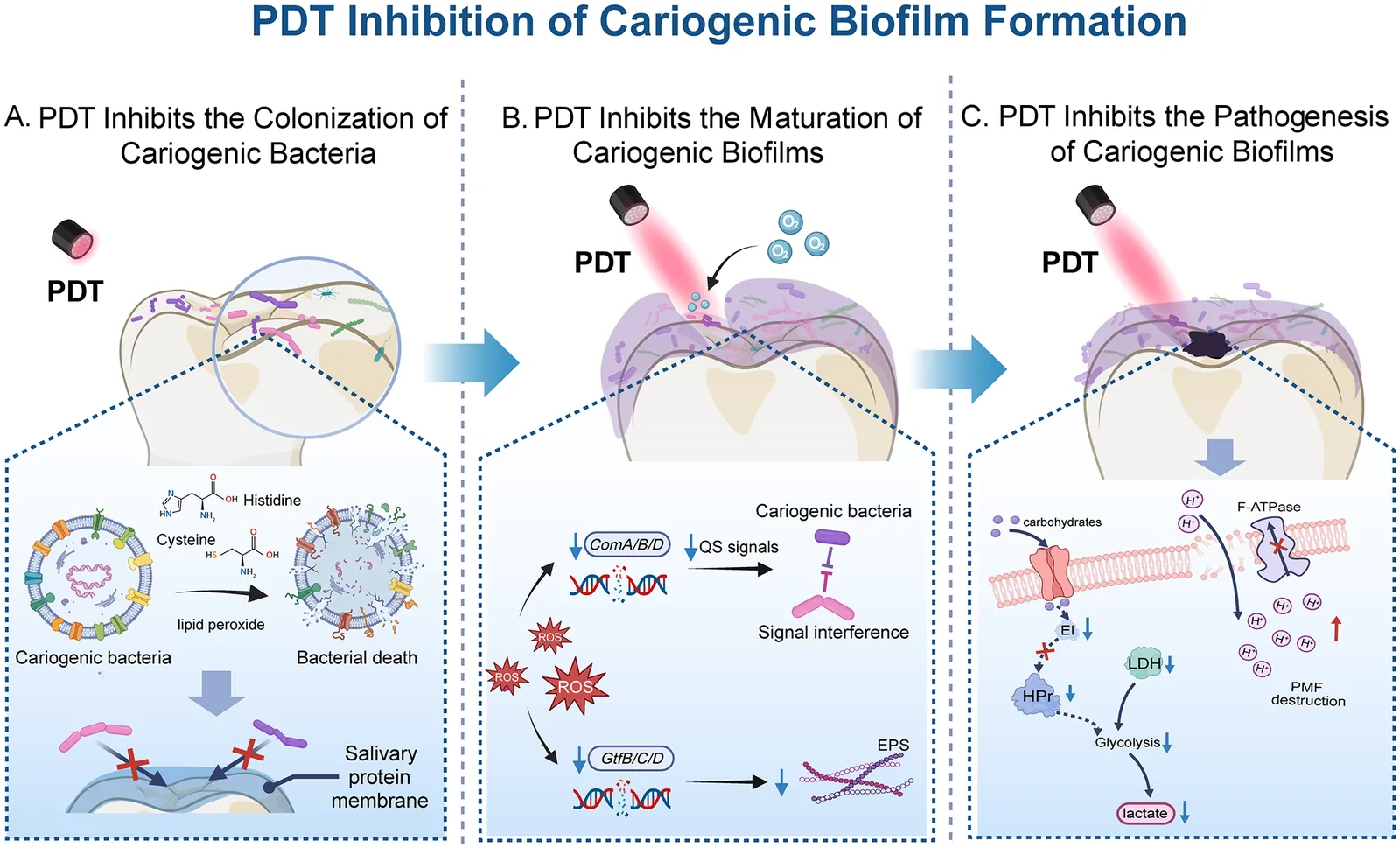
PDT inhibition of cariogenic biofilm formation: **(A)** PDT inhibits the colonization of cariogenic bacteria on salivary protein pellicles via bactericidal effects; **(B)** PDT inhibits the maturation of cariogenic biofilms by interfering with QS signals and disrupting EPS formation; **(C)** PDT inhibits the pathogenesis of cariogenic biofilms by blocking glycolysis and disrupting proton efflux channels.

#### 3.1.1 PDT inhibits the colonization of cariogenic bacteria

The colonization of cariogenic bacteria follows a chronological sequence to establish a complex microbial community. Early gram-positive (G^+^) bacteria, including *S. mutans*, first bind to the acquired enamel pellicle via surface adhesion proteins. Subsequent G^−^ bacteria attach to the surface or gaps of pioneer-originated biofilms (Kolenbrander et al., 2010). PDT oxidatively damages bacterial membrane proteins and lipids and may impair oxidative stress adaptation, thereby reducing the initial colonization of the acquired pellicle by cariogenic bacteria (Cieplik et al., 2018; Muehler et al., 2022).

PDT induces oxidative damage in bacterial membrane proteins. Initially, ^1^O_2_ attacks surface-exposed aromatic amino acid residues, such as tyrosine and histidine, as well as the sulfhydryl groups (-SH) of cysteine, directly disrupting the protein conformation (Dosselli et al., 2012). Meanwhile, ^·^OH may oxidize methionine and tryptophan residues in membrane proteins, leading to polypeptide chain cleavage or abnormal aggregation. Integral membrane proteins embedded in lipid bilayers may be particularly susceptible to damage (Clark et al., 2003).

PDT causes oxidative damage to membrane lipids. The ^1^O_2_ generated by PDT is highly electrophilic and can directly add to the unsaturated double bonds in bacterial membrane fatty acids, initiating lipid peroxidation chain reactions and generating lipid peroxides (Vatansever et al., 2013; Juan et al., 2021). The accumulation of lipid peroxides causes phospholipid cleavage, abnormal cross-linking, and disruption of bilayer organization, shifting the membrane from an ordered to a looser structure. Consequently, membrane fluidity is lost, and ion permeability increases abnormally (Angeloni et al., 2015; Herbet et al., 2017). Damage to membrane lipids reduces the stability of cariogenic bacterial adhesion to tooth surfaces and enhances drug penetration. In addition, ^·^OH generated by PDT abstracts allylic hydrogen atoms from unsaturated phospholipids in the plasma membrane, promoting their degradation and ultimately causing loss of membrane integrity and structural rupture, thereby inducing lethal damage in cariogenic bacteria (Cui et al., 2024a).

PDT disrupts intracellular redox homeostasis in bacteria. PDT rapidly generates high levels of reactive oxygen species (ROS), and when the bacterial antioxidant buffering capacity is exceeded, oxidative stress defense and repair pathways are dysregulated (Dosselli et al., 2012; Muehler et al., 2020, 2024; de Oliveira Silva et al., 2024). This may disturb the homeostasis of NADH oxidase, superoxide dismutase (SOD), and regulatory networks involving Spx, PerR, and Rex in cariogenic bacteria (Derr et al., 2012; Yu et al., 2023). This induces oxidative stress–mediated bacterial death and reduces colonization capacity.

The differential bactericidal efficacy of PDT against G^+^ and G^−^ bacteria stems primarily from the distinct physicochemical properties of their cell walls. G^+^ bacteria are generally more susceptible to PDT because they lack an outer membrane barrier, and their thick, porous peptidoglycan layer facilitates rapid PS penetration and accumulation at the cytoplasmic membrane (Pereira et al., 2014). In contrast, the efficacy of PDT against G^−^ bacteria is limited by their complex lipopolysaccharide (LPS) outer membrane (Nagata et al., 2012; Vatansever et al., 2013). The lipid A component of LPS forms a dense hydrophobic barrier, and Ca²^+^ and Mg²^+^-mediated cross-linking further restricts membrane permeability, impeding the entry of neutral or negatively charged PSs (Cantelli et al., 2020). In addition, anionic PSs may be electrostatically repelled by negatively charged phospholipids in the outer membrane of G^−^ bacteria, thereby reducing PS diffusion and antibacterial efficacy (George et al., 2009; Sułek et al., 2020). Accordingly, strategies to improve PDT against G^−^ bacteria focus on disrupting the integrity of outer membrane. Cationic PSs can competitively displace Ca²^+^ and Mg²^+^, destabilizing LPS and increasing membrane permeability. Combining PSs with outer membrane-disrupting agents can further enhance the photoinactivation of G^−^ bacteria.

PDT works by applying PS topically and exposing the area to light, with its antibacterial effect precisely confined to the affected area, thereby avoiding damage to surrounding healthy tissue. In addition, this therapy primarily targets the plasma membrane of cariogenic bacteria. PS does not need to enter bacterial cells and can kill bacteria upon light activation. As its bactericidal action relies on transient nonspecific oxidative damage, cariogenic bacteria rarely develop resistance, effectively addressing the limitations of traditional antimicrobial agents (Zanin et al., 2005). Building on this advantage, we can improve PDT efficacy against cariogenic bacteria and biofilms by optimizing PS targeting plasma membrane proteins and lipids or by incorporating permeability enhancers and efflux pump inhibitors into PS (Shrestha and Kishen, 2014).

#### 3.1.2 PDT inhibits the maturation of cariogenic biofilms

During cariogenic biofilm maturation, cariogenic bacteria coordinate collective behaviors through quorum sensing (QS) systems, produce EPS that provide structural support, and maintain a cariogenic microenvironment characterized by deep-layer anaerobiosis and sustained acidification (Li et al., 2002; Bowen et al., 2018; Ishkov et al., 2020; Kaspar et al., 2021). PDT inhibits biofilm formation by disrupting QS signaling, damaging EPS, and disturbing microenvironmental homeostasis.

PDT inhibits cariogenic biofilm formation by disrupting the biosynthesis of QS systems in cariogenic bacteria. The CSP/ComD–ComE and LuxS/AI-2 systems are the major QS pathways in these bacteria (Kashi et al., 2025). Studies have shown that PDT and certain PSs can downregulate QS-related genes, including *comA*, *comB*, *comDE*, and *luxS* (Li et al., 2019; Pourhajibagher et al., 2022a; Ghanemi et al., 2023). This transcriptional suppression might reduce QS signal secretion, thereby inhibiting cariogenic biofilm maturation. Interference with QS signaling may also decrease the ecological dominance of cariogenic bacteria and promote the restoration of microbial community homeostasis.

PDT inhibits cariogenic biofilm formation by suppressing EPS biosynthesis. At the transcriptional level, PDT downregulates the genes involved in glucan and fructan synthesis, including *gtfB*, *gtfC*, *gtfD*, and *ftf*, and affects the expression of genes related to glucan binding and adhesion, such as *gbpA* (Li et al., 2019, 2024; Pourhajibagher et al., 2022b; Ghanemi et al., 2023; Kashi et al., 2025). These effects may limit the synthesis and secretion of EPS. PDT can also reduce the EPS content of *S. mutans* biofilms and weaken their structural stability, thereby disrupting biofilm architecture (Nemezio et al., 2017; Misba et al., 2018).

PDT may also affect the oxygen gradients within cariogenic biofilms. Because both Type I and Type II photodynamic reactions are highly oxygen-dependent, excited-state PSs compete strongly for oxygen with bacteria in the superficial biofilm layers (Cieplik et al., 2018; Xiu et al., 2022). This oxygen competition may suppress aerobic glycolysis in surface-layer bacteria. Therefore, disruption of the intra-biofilm oxygen gradient may hinder the development of dense and stable mature biofilm structures. However, this proposed mechanism requires further experimental validation.

Although PDT has considerable potential for inhibiting cariogenic biofilm maturation, the treatment of highly mature and dense plaque biofilms remains challenging. As its antimicrobial action is highly oxygen-dependent, hypoxic or anaerobic conditions in the deeper layers of mature cariogenic biofilms can reduce its efficacy. To address this limitation, oxygen-carrying systems may enable the codelivery of PSs and oxygen (Xiu et al., 2022; Xu et al., 2024), thereby improving oxygen availability in deeper biofilm regions, enhancing the disruption of mature cariogenic biofilms, and optimizing PDT performance.

#### 3.1.3 PDT inhibits the pathogenesis of cariogenic biofilms

During the metabolically active phase of cariogenic biofilms, cariogenic bacteria take up carbohydrates via the phosphotransferase system (PTS) and convert them into lactic acid via glycolysis. They also rely on F-ATPase-mediated proton extrusion to maintain an acid-tolerant phenotype, contributing to the destruction of hard dental tissues (Ajdic and Chen, 2013; Lemos et al., 2019; Sekiya et al., 2023). PDT reduces acid production by decreasing the cariogenic bacterial load, inhibiting carbohydrate metabolism, and impairing proton extrusion.

PDT alters acid production in cariogenic bacteria by interfering with their carbohydrate uptake and glycolysis. At the carbohydrate uptake level, PDT may impair PTS-mediated transport, thereby weakening energy metabolism. Proteomic analysis has shown that PDT reduces the levels of the PTS proteins EI (PtsI) and HPr (PtsH) in *Staphylococcus aureus*, accompanied by decreased glucose consumption (Dosselli et al., 2012). These findings suggest that PDT-induced oxidative stress impairs PTS function and reduces carbohydrate uptake and utilization in bacteria. PDT may suppress glycolysis in cariogenic bacteria by reducing the abundance of glycolytic enzymes. PDT significantly decreases the protein levels of several glycolytic enzymes, including GAPDH, Pgk, and LDH (Dosselli et al., 2012). Together, these changes may reduce carbohydrate metabolism and limit LDH-catalyzed conversion of pyruvate to lactate, ultimately decreasing the production of lactic acid and other acidic metabolites.

The ROS generated by PDT may also impair the activity of virulence-associated enzymes, thereby reducing the production of acidic metabolites. ROS oxidizes the catalytic cysteine residue of GAPDH, resulting in enzyme inhibition (Deng et al., 2014). LDH may also form crosslinked protein aggregates after PDT, potentially compromising its structural integrity and catalytic activity (Dosselli et al., 2012; Alves et al., 2014). In addition, ROS can alter the conformation and oligomeric state of molecular chaperones, such as GroEL, thereby disrupting protein folding and stress adaptation (Melkani et al., 2004). PDT may reduce the virulence of cariogenic bacteria through these mechanisms. However, direct evidence is currently mainly derived from *S. aureus* and other model bacteria, and further validation in cariogenic species is required.

In addition to suppressing acid production, PDT may impair proton extrusion, thereby reducing the acid tolerance of cariogenic bacteria. PDT-induced membrane damage increases proton permeability, promotes proton influx, and dissipates proton motive force (PMF) (Yang et al., 2023). PMF supports membrane energy coupling and intracellular pH homeostasis; therefore, its dissipation weakens bacterial resistance to acid stress (Yang et al., 2023). PMF disruption may also permit proton backflow through transmembrane pathways, reducing the proton extrusion efficiency of F-ATPase and further disturbing intracellular homeostasis.

### 3.2 PDT promotion of remineralization and tissue repair

During the defensive and reparative phases of dental caries, PDT may exert dual effects through photobiomodulation (PBM) and low-level ROS signaling. This may promote host immune regulation and create a favorable microenvironment for dental hard tissue regeneration. It may also stimulate the pulp–dentin complex to form reparative dentin and act synergistically with mineralization-promoting agents to enhance remineralization, thereby accelerating the structural restoration of the dental tissues (**Figure 2**).

**Figure 2:**
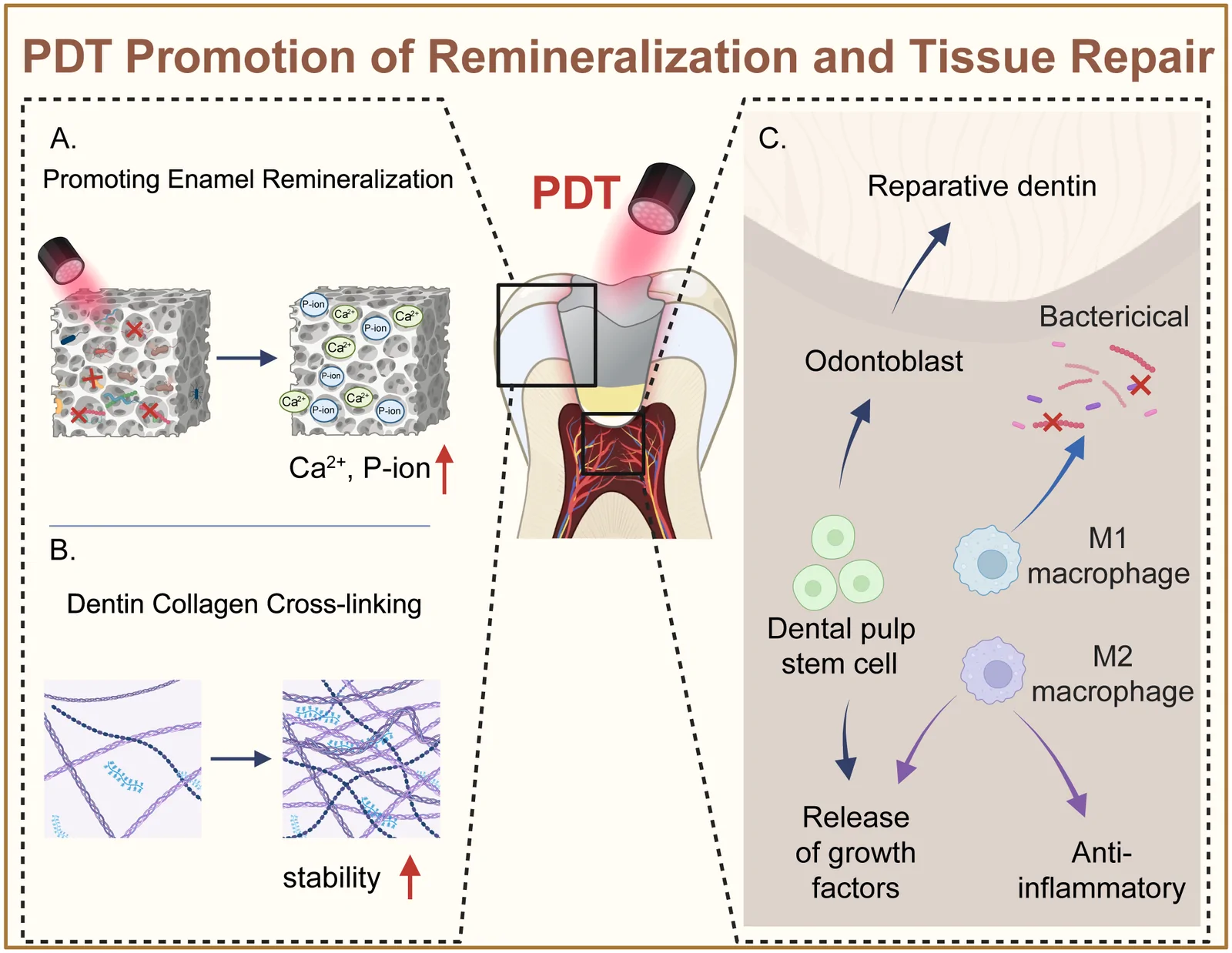
PDT Promotion of Remineralization and Tissue Repair: **(A)** PDT facilitates the deposition of calcium and phosphate ions to promote enamel remineralization; **(B)** PDT induces dentin collagen cross_linking to enhance dentin hardness; **(C)** PDT regulates the biological functions of dental pulp immune cells and dental pulp stem cells (DPSCs).

#### 3.2.1 PDT promotion of dental hard tissue repair

PDT may promote dental hard tissue repair, mainly by enhancing enamel remineralization, inducing photochemical cross-linking of the dentin organic matrix, and increasing tissue hardness.

Laser irradiation used in PDT may promote enamel remineralization through multiple mechanisms (Nammour et al., 2005; Al-Maliky et al., 2020). It can induce physicochemical modifications in the enamel by altering crystal surface polarization and improving surface micromorphology, thereby facilitating the retention and adhesion of mineral ions. Laser treatment may also promote pyrophosphate formation on the enamel surfaces and enhance acid resistance (Nammour et al., 2025b). Localized heat generated at the fiber tip may further increase enamel microhardness (Abd El-Aal et al., 2024; Nammour et al., 2025b). Overall, PDT may support enamel remineralization through physicochemical modifications.

In addition, ROS generated by PDT may promote photooxidative cross-linking of dentin collagen. Tyrosine residues (T–H) in collagen can undergo photooxidation to form tyrosyl radicals (T•), which dimerize into dityrosine (T–T), thereby cross-linking the collagen matrix (Kato et al., 1994). The resulting denser collagen network shows greater stability and resistance to degradation, and under specific conditions, may increase dentin microhardness and elastic modulus (Shrestha et al., 2011; Fawzy et al., 2012).

#### 3.2.2 PDT promotion of pulp-dentin complex repair

PDT may promote reparative dentin formation by the pulp–dentin complex and activate the intrinsic healing potential of the dental pulp, providing an active approach for tissue repair.

PDT may influence the proliferation and odontoblastic differentiation of dental pulp stem cells (DPSCs) through the photobiomodulatory effects of light components (Yong et al., 2023). Red or near-infrared light at 600–810 nm, commonly used in PDT, can penetrate dental tissues in deep carious lesions and reach the pulp, while also exerting intrinsic PBM effects. Complex IV of the mitochondrial respiratory chain acts as the primary photon receptor (de Freitas and Hamblin, 2016). Light absorption accelerates electron transport, increases mitochondrial membrane potential, and enhances ATP synthesis (Malekpour et al., 2024). Thus, PDT-associated irradiation may provide energy support for DPSC proliferation and differentiation, while promoting matrix synthesis and mineralization-related activity.

PDT may promote reparative dentin formation by modulating DPSCs. Evidence from *in vitro* and *in vivo* studies suggests that PDT-generated ROS may act as signaling molecules and activate redox-sensitive pathways, including Nrf2 and MAPK, thereby promoting the differentiation of DPSCs into odontoblast-like cells (Zaccara et al., 2020; Yong et al., 2023). Photobiomodulation studies have further indicated that low-level light can regulate the secretion of factors such as TGF-β and VEGF by DPSCs, creating a favorable environment for the repair and regeneration of injured pulp tissue (Arany et al., 2014; Sadeghi et al., 2026). These findings provide indirect support for the regulation of paracrine signaling in pulp cells through the light-related effects of PDT. One study identified an organized odontoblast layer and cellular processes extending into the dentinal tubules in PDT-treated regenerated tissue, suggesting that PDT may not only induce reparative dentin deposition but also support functional regeneration of the pulp–dentin complex (Takahashi et al., 2023). Histological sections also showed reduced inflammatory cell infiltration, supporting the combined immunomodulatory and hard tissue reparative effects of PDT.

#### 3.2.3 PDT optimization of the reparative microenvironment

During the defensive and reparative phases of dental caries, PDT may exert multidimensional immunomodulatory effects by enhancing infection clearance and activating anti-inflammatory mechanisms, thereby creating a microenvironment that is favorable for dental hard-tissue regeneration.

PDT may enhance host immune clearance of cariogenic bacteria. It can directly reduce the local cariogenic bacterial burden, thereby limiting the persistent stimulation of pulp tissue by bacterial components and metabolites, and creating conditions favorable for tissue recovery. In tumor and skin models, PDT has been shown to activate local immune cells, promote the release of inflammatory mediators, and enhance phagocytic and cytotoxic activities (Yu et al., 2021, 2022; Bahrami et al., 2024). Thus, in caries-affected pulp tissue, the reduction in the bacterial burden induced by PDT may act together with an early immune response to promote the clearance of residual pathogenic components. However, because direct evidence of M1 polarization and related cytokine changes in pulp PDT models is lacking, this mechanism is currently speculative.

PDT initiates an anti-inflammatory response. Studies in wound healing models have shown that PDT can promote macrophage polarization toward the M2 phenotype, accompanied by an increased expression of anti-inflammatory cytokines such as IL-10 (Cui et al., 2024b). Temporal regulation of macrophage polarization promotes dentin regeneration, and PDT exhibits anti-inflammatory effects (Li et al., 2021; Kadowaki et al., 2022). It is reasonable to infer that PDT may also promote M2 macrophage polarization in dental pulp, thereby creating an anti-inflammatory microenvironment favorable for pulp tissue repair.

## 4 Clinical applications of PDT in caries management

### 4.1 PDT applications in caries prevention

Primary prevention of dental caries currently relies on mechanical plaque removal, topical fluorides, including fluoride varnish, and pit-and-fissure sealants (Marinho et al., 2003, 2013). These measures are clinically well-established, promote remineralization, and reduce plaque retention; however, their effectiveness depends heavily on long-term patient adherence. Among orthodontic patients, individuals with xerostomia, and those requiring special care, conventional oral hygiene is often insufficient to control local biofilms. Antimicrobial agents, such as chlorhexidine, may serve as adjuncts; however, their beneficial effects remain inconsistent, and prolonged use may disrupt the oral microbiota and cause tooth staining (Walsh et al., 2015; James et al., 2017).

PDT, mechanical plaque removal, and antimicrobial agents can reduce cariogenic bacterial load and inhibit cariogenic biofilms. However, PDT cannot replace mechanical plaque removal and is, therefore, better positioned as an adjunct to individualized caries prevention. Its noninvasive nature and the absence of substantial noise or vibration may improve its acceptance among children and other patients with dental anxiety. Beyond precise local application in clinical settings, PS mouth rinses combined with portable light sources may offer a more convenient approach for whole-mouth biofilm control (Shi et al., 2025). In addition, the combined application of PDT and whitening treatments warrants further exploration to expand the scope and value of PDT applications. Currently, it may be particularly relevant for high-risk populations, including orthodontic patients, individuals with xerostomia, children with poor adherence, and patients with intellectual disabilities or other special care needs (Paschoal et al., 2015; Gong et al., 2019; Kamran et al., 2021; Morozova et al., 2023; Shafaee et al., 2023; Barrameda et al., 2025).

PDT aims to suppress early-colonizing G^+^ cariogenic bacteria such as *S. mutans* and shift the oral microbiome from a cariogenic to a healthier state. Its primary role is to prevent the formation of cariogenic biofilms, thereby disrupting the acid cycle. PDT has shown clear inhibitory effects against *S. mutans* because early biofilms are relatively thin, and conventional PSs generally exhibit broad applicability and sensitivity (Ishiyama et al., 2012; Hirose et al., 2021; Terra-Garcia et al., 2021). Given the predominance of G^+^ bacteria at this stage, lipophilic PSs, such as curcumin, may be preferred (Erckmann et al., 2025). Curcumin activated by a 450-nm LED may selectively target G^+^ bacteria and help restore the oral microbial balance. Curcumin-mediated PDT can also downregulate pro-inflammatory mediators, providing additional benefits by alleviating gingival inflammation and xerostomia-associated mucosal injury while preventing caries (Ahrari et al., 2023; Afrasiabi et al., 2024; Erckmann et al., 2025). For patients with xerostomia, whose biofilms often contain bacterial-fungal communities, PSs with strong penetration and antifungal activity should also be considered (Vieira Mendes et al., 2025) (**Table 1**).

**Table 1:** Photodynamic therapy for caries prevention.

Photosensitizer class	Representative PSs	Research models	Results	Mechanism	Author (Year)
Phenothiazines	MB	*In vivo*: Children dentin caries; *In vitro*: Artificial dentin caries; *S. mutans* (planktonic/biofilm)	Bactericidal	Cationic: Direct membrane damage	(Neves et al., 2016; Nagai et al., 2018; Alves et al., 2019; Azizi et al., 2019; Shojaeian et al., 2024)
	PapaMBlue (Enzyme complex)	*In vivo*: Children dentin caries; *In vitro*: *S. mutans* (planktonic/biofilm); Mixed cariogenic flora	Reduces biofilm; Synergistic antibiosis; Minimally excavation	Inhibits dimerization; Softens carious debris; Boost ^·^OH yield	(Silva et al., 2016; Vieira et al., 2019; Costa-Santos et al., 2019)
	MB acid combination	*In vitro*: Artificial dentin caries	Synergistic antibiosis; Simplify procedures	Acidic microenvironment	(Pinheiro et al., 2019)
	MB-NPs	*In vitro*: *S. mutans* (planktonic/biofilm); *L. acidophilus* (biofilm)	Reduces biofilm; Removes smear layer	Inhibits dimerization; Boost ROS yield; Improve solubility; Cationic: Direct membrane damage	(Shahmoradi et al., 2022; Binhasan et al., 2023; Alanazi et al., 2024b)
	TBO	*In vivo*: Saliva of S-ECC; Clinical *S. mutans* suspension; *In vitro*: Artificial dentin caries; *S. mutans* (planktonic/biofilm)	Bactericidal	Cationic: Direct membrane damage	(de Melo et al., 2013; Bargrizan et al., 2019; Barrameda et al., 2025; Karamad et al., 2025)
	NLITBO	*In vitro*: *S. mutans* (planktonic)	Bactericidal; Sustained release	Liposome: Enhances intracellular penetration; Direct membrane damage	(Panda et al., 2024)
Cyanine Dyes	ICG	*In vitro*: *S. mutans*/*L. acidophilus* (planktonic)	High penetration	Photothermal damage; EPS penetration	(Azizi et al., 2016; Ahrari et al., 2018; Aytac Bal et al., 2019)
Chlorins	FTC/FDZ	*In vitro*: *S. mutans* (planktonic/biofilm); Mixed cariogenic flora	Bactericidal; High solubility	Cationic: Direct membrane damage; Acid inhibition	(Terra Garcia et al., 2018; Garcia et al., 2021; Terra-Garcia et al., 2021)
Xanthenes	Rose Bengal/CSRBnps	*In vitro*: Mixed cariogenic flora; *L. acidophilus* (planktonic)	Plaque staining; CSRBnps: Synergistic antibiosis	CSRBnps: Cationic: Direct membrane damage; Inhibits ACQ; Sustained ROS release	(Bulit et al., 2014; Carvalho et al., 2020; Hirose et al., 2021)
Polyphenols	Curcumin/Nano-curcumin	*In vitro*: *S. mutans*/*L. acidophilus* (planktonic/biofilm)	High biocompatibility; Low price; Nano-curcumin: Sustained action	Anti-inflammatory; Nano-curcumin: Improve solubility	(Araújo et al., 2014, 2017; Manoil et al., 2014; Azizi et al., 2019; Ahrari et al., 2023; Alkhudhairy and Aljamhan, 2023; Afrasiabi et al., 2024; Erckmann et al., 2025)
Phthalocyanines	ZnPcS4	*In vitro*: *S. mutans* (biofilm)	Reduces biofilm; Enhances efficacy	Improved Solubility; Self-Oxygenation	(Zhang et al., 2024)
AIEgens	Triphenylamine	*In vitro*: *S. mutans* (planktonic/biofilm);	Adhesion to biofilms; Enhances efficacy; Remineralization; Tooth whitening	Interacts electrostatically; Self-Oxygenation; Inhibits ACQ	(Cao et al., 2024)
Inorganic Materials	TiO_2_/HAP	*In vivo*: *S. mutans* (Planktonic/Biofilm); Rat caries model	Reduces biofilm; Remineralization; Tooth whitening	Biomineralization; Photocatalysis	(Wang et al., 2025)

### 4.2 PDT applications in non-operative treatment of dental caries

Current non-operative treatments for dental caries include topical fluorides, silver diamine fluoride (SDF), and resin infiltration (Gao et al., 2016; Elrashid et al., 2019). Fluorides and SDF promote remineralization and inhibit the progression of active carious lesions, whereas resin infiltration delays lesion progression by sealing the pores in early enamel caries. However, SDF can cause black staining of lesions, limiting its use in esthetically sensitive areas. PDT may serve as an adjunct to conventional non-operative treatment by reducing cariogenic bacterial loads in lesions, increasing enamel microhardness, and enhancing calcium and phosphate ion deposition on demineralized enamel (Bahrololoomi and Lotfian, 2015; Alshamrani, 2023). Advances in photoresponsive bioactive materials may integrate antibacterial, remineralizing, and whitening effects with PDT, thus providing a minimally invasive strategy for the treatment of early caries, white spot lesions (WSLs), and tooth discoloration. Clinically, PDT is mainly used for the remineralization management of early enamel caries and WSLs, as well as for minimally invasive intervention for early childhood caries (ECC), offering a non-invasive therapeutic option for high-risk populations (Bargrizan et al., 2019; Gong et al., 2019; Shafaee et al., 2023; Bahrami et al., 2024; Lamorgese et al., 2025).

The ability of PDT to increase enamel microhardness may be useful in primary teeth and early carious lesions, where the enamel is less mineralized and more susceptible to erosion. Treatment with 100 μg/mL MB and a 660-nm laser significantly increased enamel microhardness, with an effect comparable to that of SDF (Valizade et al., 2024). This enhancement may result from the local photothermal effects of diode laser irradiation, which induces microstructural reorganization of the enamel surface crystals (Hashemikamangar et al., 2024; Nammour et al., 2025a). Thus, PDT may provide a non-pharmacological approach for strengthening demineralized enamel. It may also offer a potentially feasible minimally invasive strategy for hard tissue defects, including amelogenesis imperfecta and dentinogenesis imperfecta in the future.

Thus, PDT may serve as an effective pretreatment that synergizes with enamel remineralization. When combined with conventional fluorides such as titanium tetrafluoride (TiF_4_) and sodium fluoride (NaF), diode laser irradiation can induce microscopic physical modifications that increase enamel surface roughness and promote calcium and phosphate deposition. The integration of PDT with nanomaterials may further enhance remineralization. Nanomicellar curcumin, propolis nanoparticles, and chitosan-based nanomaterials have been reported to improve the rate and long-term stability of remineralization (Zaleh et al., 2022). PDT can also be combined with bioactive materials, including titanium oxide (TiO_2_)/hydroxyapatite (HAP) nanocomposites and curcumin-loaded zein nanocapsules (Lu et al., 2023; Erckmann et al., 2025; Wang et al., 2025). These materials can function as heterogeneous nucleation templates that mimic amelogenin by providing an organic scaffold for apatite deposition and guiding remineralization. In summary, by integrating antibacterial, remineralizing, and whitening effects, PDT offers a comprehensive and minimally invasive approach for managing early caries, WSLs, and tooth discoloration (**Table 2**).

**Table 2:** Photodynamic therapy for non-operative caries treatment.

Photosensitizer class	Representative PSs	Research models	Results	Mechanism	Author (Year)
Phenothiazines	TBO (Fluoride Combination)	*In vitro*: Human enamel discs; Demineralization model	Simultaneous disinfection & remineralization; High acid resistance	Melting & recrystallization; Increased porosity; Phosphate binding; Elevated Ca/P ratio	(Hashemikamangar et al., 2024; Moradi et al., 2024)
	MB	*In vitro*: Demineralization model	High hardness;	Phosphate binding	(Valizade et al., 2024)
Polyphenols	Curcumin NCs	*In vitro*: Human dentin discs	Sustained release; Anti-inflammatory	Mineralization via endogenous Ca-P Ions	(Erckmann et al., 2025)
	Nano-curcumin/NPro combination	*In vitro*: Bovine enamel	Rapid onset; High hardness	NPro: Direct Ca/P release; Melting & recrystallization	(Zaleh et al., 2022)
Inorganic Materials	TiO_2_/HAP	*In vivo*: Demineralization model; Tooth staining & whitening model	High Hardness; Tooth Whitening	HAP: Direct Ca/P Release	(Wang et al., 2025)

### 4.3 PDT applications in operative treatment of dental caries

Minimally invasive approaches remain the mainstay of operative treatment for dental caries. PDT may further reduce the microbial load in the residual dentin and dentinal tubules without additional removal of the tooth structure (Cieplik et al., 2017; Aytac Bal et al., 2019; Kuderewska et al., 2026; Schwendicke et al., 2026). Thus, controlling residual microorganisms and achieving an adequate restoration seal are critical for treatment outcomes. Clinically, PDT may be used as an adjunctive disinfection method after chemomechanical or selective caries removal and combined with adhesive restoration under reliable sealing conditions (AlSunbul and Murriky, 2023; ElMourad and Al-Shamrani, 2024; Almutairi, 2025). PSs can be incorporated into etchants, adhesives, and chemomechanical caries-removing agents. This approach integrates etching, disinfection, and bonding into a single step while maximizing the preservation of sound hard tissue, thereby enabling more intelligent and minimally invasive restorative systems (Pinheiro et al., 2019; Vieira et al., 2019; Hashem, 2021; Lazarova et al., 2026).

PDT has substantial clinical potential for eliminating *Lactobacillus* spp., which predominate in deep carious lesions (Araújo et al., 2014; Ahrari et al., 2023; Alkhudhairy and Aljamhan, 2023; Baghani et al., 2024). As *L. acidophilus* tends to colonize deep dentinal tubules, conventional chemical irrigants have limited penetration and fail to completely eradicate latent infection (Nadkarni et al., 2010). In contrast, PSs can penetrate most of the infected depth. Subsequent activation with deeply penetrating diode lasers, such as 660 and 810 nm lasers, can significantly reduce bacterial density within the dentinal tubules (Nogueira et al., 2013; Savadi Oskoee et al., 2013). Combining PDT with sonodynamic-photodynamic therapy (snoPDT) may further improve PS delivery into the dentinal tubules through ultrasonic cavitation, thereby enhancing deep disinfection (Alkhudhairy, 2025; Sharma et al., 2025). In addition, ICG activated by an 810-nm laser provides favorable near-infrared penetration and may be a preferred adjunctive approach for photoinactivation of cariogenic microorganisms in deep caries management.

To guarantee bonding interface stability, clinicians can select a self-etch adhesive system with better biochemical compatibility with the treated surfaces. This is because the total-etch system fully opens the dentinal tubules. Consequently, the ROS produced by PDT may interfere with the integration of the adhesive and resin (Marimoto et al., 2013; Sekhar et al., 2017; Nassaj et al., 2020; Rodriguez et al., 2025). The PS selection may vary depending on the bonding substrate used. In enamel, most PSs do not affect bond strength; thus, selection is not strictly limited (Madani et al., 2018; Nassaj et al., 2020). Riboflavin and curcumin may have more stable effects than MB in caries-affected dentin (CAD). PSs with collagen cross-linking activity, such as riboflavin and curcumin, can induce chemical cross-linking of dentin collagen fibers and enhance the long-term stability of the bonding interface (Fawzy et al., 2012; Seseogullari-Dirihan et al., 2015; Thummala et al., 2025). In contrast, MB, TBO, and high concentrations of PSs may leave residual ROS at the interface and reduce the shear bond strength (Nassaj et al., 2020; Keskin et al., 2021; Saffarpour et al., 2022; Mendes et al., 2024). ICG is preferred for root caries or cementum margins (Saffarpour et al., 2022). To mitigate the potential adhesive interface contamination caused by PDT, the combined application of chitosan is recommended. This strategy enables calcium and phosphate ions to induce crystal nucleation on the dentin surface, counteracts the detrimental effects of ROS, and expands the clinical scope of available PSs (Alanazi et al., 2024a; Mendes et al., 2024).

### 4.4 PDT applications in vital pulp therapy

However, the application of PDT in vital pulp therapy (VPT) remains to be explored. PDT may exert dual antibacterial and anti-inflammatory effects, optimizing the local microenvironment. In addition, PBM may activate DPSCs to induce reparative dentin formation in the pulp cavity. This may offer a minimally invasive strategy for treating high-risk deep caries and potentially improve the success rate of VPT (Guglielmi et al., 2011; Sharma et al., 2014; Elchaghaby et al., 2020; Fouad et al., 2024).

Through its combined antibacterial and anti-inflammatory effects, PDT may help reduce microbial burden and inflammation, thereby creating a local microenvironment that supports successful pulp capping. Its efficacy in eliminating pathogenic microorganisms at the pulp-capping sites is well supported (Bulit et al., 2014; Melo et al., 2015). PDT has also shown photobiomodulatory effects in periodontal therapy, root canal treatment, and pulpotomy, including reduced pro-inflammatory cytokine levels and postoperative pain (Afkhami et al., 2023; Bahrami et al., 2024; Das et al., 2025). Accordingly, it can be inferred that PDT is also capable of reducing pro-inflammatory mediators within the pulp tissues of deep caries. Future studies should combine *in vitro* inflammatory models of DPSCs, animal caries-pulpitis models, and clinical case-control studies. Testing canonical pro-inflammatory mediators will fully validate the local anti-inflammatory activity of PDT.

The ability of PDT to induce reparative dentin formation may be relevant to VPT. *In vitro* studies have demonstrated that PDT promotes differentiation of DPSCs into an odontoblast-like lineage (Arany et al., 2014; Li et al., 2020; Asnaashari et al., 2022). Moreover, PDT may exert prominent synergistic effects with pulp-capping materials, including mineral trioxide aggregate (MTA), biodentine, and calcium hydroxide (Banci et al., 2020; Zafari et al., 2022). This may be associated with the enhanced formation and deposition of reparative dentin, potentially contributing to more favorable long-term outcomes of pulp capping therapy. Some *in vivo* studies have reported that, following root canal disinfection with PDT during regenerative endodontic treatment, newly formed tissues may exhibit relatively organized dentinal tubule-like structures (Takahashi et al., 2023). This may be attributed to the thorough bacterial elimination and pro-healing effects of PDT on dental pulp tissues, as mentioned above.

In VPT, PDT must be performed with strict biosafety parameters to avoid pulpal damage. Clinically, low-dose strategies are recommended (Diniz et al., 2014; Shojaeian et al., 2024). Low-dose activation with red to near-infrared light (660–810 nm), with strict control of the energy density and output power, may have photobiomodulatory effects (Zaccara et al., 2020). For MB-mediated PDT, a conservative regimen may use 0.0125 mg/mL MB, a 5-min incubation period, and 660-nm red-light irradiation (40 mW for 60 s; 2.4 J; 60 J/cm²). However, its long-term safety and clinical benefits require confirmation through high-quality studies (Diniz et al., 2014, 2015). Intermittent irradiation, enhanced heat dissipation, and shorter exposure times may mitigate cytotoxicity associated with high PS concentrations, pulpal injury caused by intrapulpal temperature increases above 5.5 °C during high-power irradiation, and ROS-induced oxidative stress (Cieplik et al., 2017). These measures may help ensure the biosafety of PDT in VPT (**Table 3**).

**Table 3:** Photodynamic therapy for vital pulp therapy.

PSs	Research models	Results	Mechanism	Author (Year)
MB/MB-NPs	Dental Pulp Cells (*In vitro/vivo*)	Concentration-dependent toxicity (<0.125mg/mL); Time-dependent toxicity (<72h); Reparative dentin formation	Pulp cell proliferation activation; DPSCs differentiation into odontoblasts; Dentin barrier against ROS;	(Diniz et al., 2014, 2015; Ferreira et al., 2022; Takahashi et al., 2023; Shojaeian et al., 2024)
	Immune Cells (*In vivo*)	Stress-induced inflammation	Laser thermal effect	(Wang et al., 2025)
TBO	Dental Pulp Cells (*In vitro*)	Time-dependent toxicity (<48h); Light Dose-dependent concentration (24J/cm²)	Cationic: Bacteria-cell selective binding	(Li et al., 2022; Wang et al., 2025)
ZHC-NSs	Dental Pulp Cells (*In vitro*)	Concentration-dependent toxicity (<2mg/mL)	Abnormally high ROS/Zn²^+^ accumulation	(Afrasiabi et al., 2025)
AlClPc	Dental Pulp Cells (*In vitro*)	Short-term incubation (5min)	Cationic: Bacteria-cell selective binding	(Longo et al., 2012)

## 5 Conclusions and perspectives

Driven by its distinct mechanism of action, PDT may serve as a non-invasive adjunctive approach for caries intervention. Under appropriate conditions, it may eliminate cariogenic biofilms and fundamentally halt caries progression. Given its antimicrobial activity and low propensity for resistance, PDT has been explored as an adjunct in several stages of caries management, from prevention to cavity preparation. This may be particularly relevant for high-risk populations and the management of pediatric caries. In hard-tissue restoration, combining PDT with remineralizing agents may provide complementary antimicrobial and remineralizing benefits, thereby potentially addressing the limitations of monotherapy. Furthermore, by modulating inflammatory responses to enhance microbial clearance and by supporting reparative dentin formation, PDT highlights its potential in vital pulp therapy and regenerative endodontics. Ultimately, the therapeutic benefits of PDT extend well beyond basic disinfection, offering a comprehensive and highly practical framework for caries management, from mechanistic research to clinical translation.

However, the application of PDT in the treatment of caries remains under development and faces key translational barriers, including inadequate protocol standardization, insufficient evidence for emerging applications, and limited clinical accessibility. A major challenge is the lack of standardized treatment parameters because studies on PDT’s antimicrobial and bonding effects have used inconsistent models and outcome measures. Therefore, parameter selection remains difficult in clinical practice, where outcomes may differ from experimental findings, and the desired therapeutic effects may not be achieved. Thus, establishing stage-specific standardized parameter systems for caries treatment is necessary to facilitate its clinical application. Long-term randomized controlled trials are needed to assess the actual clinical efficacy of PDT. PDT also has potential value in caries remineralization and vital pulp therapy; however, the evidence supporting these applications remains limited. Further *in vivo* studies are needed to clarify the conditions under which PDT can achieve favorable remineralization and vital pulp preservation outcomes, thereby providing a basis for clinical use. Meanwhile, clinical accessibility is constrained by the high cost of PDT equipment, complex maintenance and training requirements, expense of PSs such as Ce6, ICG, emerging nano-delivery systems, and the need for laser activation in some protocols. The development of low-cost PSs and LED-compatible irradiation protocols may improve accessibility. Only after its safety, restorative compatibility, long-term efficacy, and clinical operability have been fully validated can PDT become a standardized clinical strategy across the caries management continuum.
